# Editor’s Choice Protein engineering strategies to develop lectins by design

**DOI:** 10.1093/glycob/cwaf041

**Published:** 2025-07-22

**Authors:** Ryoma Hombu, Lauren E Beatty, Sriram Neelamegham

**Affiliations:** Chemical and Biological Engineering, University at Buffalo, State University of New York, Buffalo, NY 14260, USA; Biomedical Engineering, University at Buffalo, State University of New York, Buffalo, NY 14260, USA; Chemical and Biological Engineering, University at Buffalo, State University of New York, Buffalo, NY 14260, USA; Biomedical Engineering, University at Buffalo, State University of New York, Buffalo, NY 14260, USA; Medicine, University at Buffalo, State University of New York, Buffalo, NY 14260, USA

**Keywords:** directed evolution, glycan, lectin, mutagenesis, surface display

## Abstract

Glycans regulate a wide array of biological processes, making them central to studies of cell biology. Thus, it is essential to characterize the spatiotemporal dynamics of glycans on cells and tissues, and to elucidate how glycan structures affect protein and cell function. Among the available molecular tools, glycan-binding proteins (GBPs), including naturally occurring lectins, are uniquely suited to provide this information at single-cell resolution. However, the diversity of cell-surface glycans far exceeds the number of readily available GBPs. Moreover, conventional lectins often possess shallow binding pockets that limit their recognition to terminal glycan epitopes, and such recognition often proceeds with low binding affinity. Protein engineering offers a promising strategy to expand GBP specificity, enhance affinity, and introduce novel binding capabilities. Currently, large gaps remain between the available protein design principles and their application to GBP engineering. This has somewhat slowed progress in the development of glycan-targeted tools. In this review, we outline recent efforts that use rational design to inform GBP engineering for specific tasks. We also present methods to select suitable protein scaffolds and the application of directed evolution for optimizing lectin design. This includes our recent efforts to modify glycosyltransferases into GBPs, which potentially offers a predictive strategy to design lectins based on desired properties. Together, the presentation offers a roadmap for developing next-generation glycan binding proteins capable of decoding the complex glycan landscape of cells.

## Introduction

Glycosylation is a ubiquitous and structurally complex post-translational modification that regulates key biological processes such as molecular recognition, cell adhesion, and signaling (Neelamegham and Mahal 2016; Varki 2017). Alterations in glycan structures often serve as biomarkers of cell differentiation and disease progression. Given these roles, profiling cell surface glycans holds significant biomedical relevance. However, such measurements are complicated by the inherent stereochemical diversity and branching patterns of glycans. Although mass spectrometry remains a powerful tool for determining glycan composition and topology—especially for abundant species—it remains challenging to differentiate structural isomers especially in complex samples, requires sample destruction, and lacks single-cell resolution. Therefore, the development of new molecular tools to characterize glycan structures is critical to advancing our understanding of functional glycobiology and to unlock its biomedical potential.

Lectins from both prokaryotic and eukaryotic sources have long been employed in biochemical assays for glycan recognition (Marathe et al. 2008; Bonnardel et al. 2021; Warkentin and Kwan 2021; Cummings 2022). These proteins are particularly valuable due to their ability to distinguish subtle structural variations in glycans, including monosaccharide stereochemistry, glycosidic bond anomericity (α vs. β) and linkage positions (e.g. α1–3 vs. α1–4). Glycan-binding proteins (GBPs) including lectins are well-suited for single-cell workflows and for detecting rare glycan epitopes that may elude detection by mass spectrometry. Despite these advantages, many lectins suffer from limited specificity due to shallow binding pockets, often resulting in promiscuous recognition of common terminal motifs shared across multiple glycoconjugates. Consequently, they typically fail to bind larger or more intricate glycan structures that serve as more specific markers of cellular transformation. Furthermore, the inherent low affinity of protein-carbohydrate interactions has contributed to the perception that “lectin binding is non-specific”. Additionally, and importantly, currently available lectins do not cover all known glycan epitopes in nature. Carbohydrate-binding modules (CBMs) are well-known GBPs that are part of glycan-processing enzymes listed at the carbohydrate-active enzyme database (CAZy (Boraston et al. 2004, Drula et al. 2022)), and these modules bring enzymes and substrates together to promote reactions. However, CBMs usually recognize glyco-homopolymers or terminal monosaccharides, and lack the ability to bind more complex structures (Armenta et al. 2017; Bonnardel et al. 2021). Anti-carbohydrate antibodies have been developed as an alternative, but low immunogenicity of carbohydrate antigens makes it challenging to derive monoclonal antibodies (mAbs) against arbitrary glycan epitopes (Haji-Ghassemi et al. 2015; Sterner et al. 2016). Additionally, the number of glycan epitopes covered by available antibodies listed at the Database of Anti-Glycan Reagents (DAGR, https://dagr.ccr.cancer.gov) is few, compared to similar reagents directed against proteins, and even the existing anti-glycan reagents may not have specific binding properties (Manimala et al. 2007).

**Figure 1:**
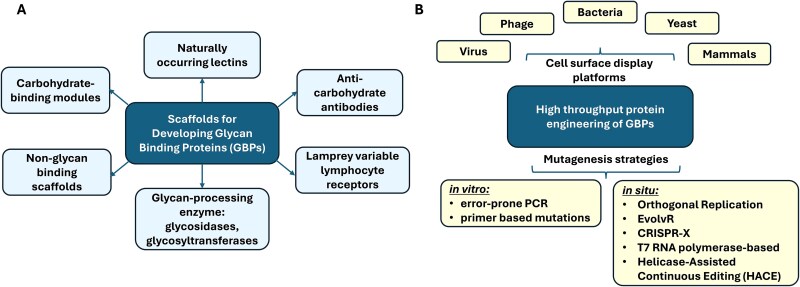
Considerations for glycan binding protein engineering: a) selection of scaffolds for developing glycan binding proteins (GBPs). b) Surface display platforms and mutagenesis strategies.

Protein engineering has recently emerged as a powerful approach for modifying nature-derived glycan-binding proteins (GBPs), as well as for repurposing non-lectin scaffolds to recognize carbohydrates (Hirabayashi and Arai 2019; Ward et al. 2021). These efforts aim both to enhance the affinity and specificity of existing protein-glycan interactions and to impart novel binding specificities to known GBPs. In this review, we systematically examine various protein scaffolds that have been used to generate or modify GBPs—including classical GBPs based on natural lectins, anti-carbohydrate antibodies, and CBMs, as well as glycan-processing enzymes and next-generation constructs (Fig. 1A). We emphasize strategies for improving the binding specificity of existing GBPs and for converting non-glycan-binding proteins into functional GBPs. Furthermore, we explore emerging techniques in GBP engineering, including the use of rational design and directed evolution paired with surface display technologies and mutagenesis across diverse host organisms (Fig. 1B). This includes our recent efforts to convert glycosyltransferases into glycan binding proteins (GBP, (Hombu et al. 2025)). Together, these strategies provide a blueprint for molecular engineering aimed at mapping the cellular glycome at single-cell resolution and tracking glycan epitope changes during biological processes and disease progression.

### Scaffold proteins for creating GBPs

The development of glycan-binding proteins (GBPs) through protein engineering requires the selection of suitable scaffold proteins. Key factors influencing scaffold suitability include the surface area of the binding pocket and the glycan diversity the scaffold can accommodate—both of which are critical for enabling recognition of the wide array of naturally occurring glycans (Ward et al. 2021; Warkentin and Kwan 2021). This section, along with Table 1, highlights a range of candidate scaffold proteins and summarizes protein engineering strategies that can be employed to optimize or expand their glycan-binding capacity.

**Table 1:** Examples of GBP engineering using lectins (top) or other scaffolds (bottom).

**Template**	**Organism**	**Protein name**	**Specificity of wild-type GBP**	**Mutagenesis method**	**3D struct. Class**	**Reference**	**Mutant property**
Lectin	Bacterial	PA-IIL	Fuc	Rational site-directed mutagenesis (crystal structure)	β-sandwich / 2 calcium lectin	(Adam et al. 2007)	Altered binding specificity to Fuc (S23A, G24N) and Man (S22A) derivatives
	Bacterial	PTL	High-Mannose	Rational site-directed mutagenesis (crystal structure)	β-barrel	(Matoba et al. 2021)	Enhanced hemagglutination and anti-influenza activity following A54N/Q62R/Q74E or L131Q
	Plant	BanLec	Man	Rational site-directed mutagenesis	β-prism I	(Swanson et al. 2015)	Decreased mitogenicity but retained antiviral activity (H84T)
	Plant	MAH	Sia	Random site-directed mutagenesis	β-sandwich / ConA-like	(Yim et al. 2001)	Increased hemagglutination activity to bovine (clone 1), equine (clone 13), chicken (clone 8, 13), and porcine (clone 3)
	Plant	PNA	Galβ1,3GalNAc (TF antigen)	Random site-directed mutagenesis	β-sandwich / ConA-like	(Soga et al. 2015)	Additional specificity to NeuAcα2,6(Galβ1,3)GalNAc in addition to original binding to Galβ1,3GalNAc (variant A)
	Fungi	ACG	lactose- or Forssman antigen	Random site-directed mutagenesis	β-sandwich / ConA-like	(Hu et al. 2013)	Binding to Forssman antigen (GalNAcα1,3Gal/GalNAc-) was retained but binding to lactose/LacNAc was lost in N46A
	Animal	E-selectin	sLe^X^	Computational modeling	α/β mixed / C-type lectin-like	(Wang et al. 2022)	Converted specificity to 6′-sulfo-sLex and 6′-sulfo-sLacNAc (E92A/E107A) similar to Siglec-8
	Animal	Galectin-1	LacNAc	Non-canonical amino acid incorporation	β-sandwich / ConA-like	(Tobola et al. 2022)	Decreased specificity to 3'-O-sulfated LacNAc and slightly increased specificity to LacNAc (CSGal-1[7FW]) compared to CSGal-1 (Galectin-1 with Ser mutations in all Cys residues)
	Animal	EW29Ch	Gal	Random mutagenesis (ribosome display)	β-trefoil	(Yabe et al. 2007)	Additional specificity to α2,6-Sia as well as the original specificity to Gal (E148G, I227N, D230G, I231V, E237G, G239S)
	Animal	EW29Ch	Gal	Random mutagenesis (ribosome display)	β -trefoil	(Hu et al. 2012)	Additional specificity to 6-sulfo-Gal as well as the original specificity to Gal (variants with E20K)
**Template**	**Organism**	**Protein name**	**Specificity of wild-type GBP**	**Mutagenesis method**	**CAZy classification**	**Reference**	**Mutant property**
CBM	Fungi	*Tl*Ga15B	Starch	Domain swapping and rational site-directed mutagenesis (mimicking stable variant, *Re*Ga15A)	CBM20	(Tong et al. 2022)	Increased expression level (domain swapped TlGa15B-M2 and M4, mutagenized TlGa15B-M6, M7)
	Fungi	Cel7A	cellulose/lignin	Rational site-directed mutagenesis	CBM28	(Strobel et al. 2016)	Increased selectivity to cellulose over lignin by negatively charged amino acid (V27E)
	Bacterial	Cel7A	cellulose/lignin	Computational modeling (Rosetta)	CBM2a	(DeChellis et al. 2024)	Increased binding to cellulose by positively charged amino acid (D4, D3-CBM2a)
	Bacterial	Xyn10A	Xyloglucan	Random site-directed mutagenesis	CBM4–2	(Gunnarsson et al. 2004)	Modified binding specificity to cellulose, mannan, human IgG4, and xylan
	Bacterial	Xyn10A	Xyloglucan	Random mutagenesis	CBM4–2	(von Schantz et al. 2009)	Modified binding specificity to xyloglucan (XG-34 1-X)

*(continued)*

**Table 1:** Continued.

**Template**	**Organism**	**Protein name**	**Specificity of wild-type GBP**	**Mutagenesis method**	**CAZy classification**	**Reference**	**Mutant property**
Glycosid-ase	Bacterial	NanB	pan-Sia	Computational modeling	GH33	(Yang et al. 2002)	Inactivated glycosidase activity and showing binding to pan-sialoglycans (Y653F, Sia-PS1)
	Bacterial	NanB	α2,3-Sia	Site-directed mutation library (yeast surface display)	GH33		Inactivated glycosidase activity and showing binding to α2,3-sialoglycans over α2,6-sialoglycan (R193A/D270Q/A538V/E541D, Sia-3S1)
	Bacterial	SpNanA	pan-Sia	Rational point mutation	GH33	(Liang et al. 2025)	Inactivated glycosidase activity and increased binding to pan-sialoglycans (D372N)
	Bacterial	RgNanH	α2,3-Sia	Rational point mutation	GH33		Inactivated glycosidase activity and increased binding to α2,3-sialoglycans (D282A)
Glycosyl-transferase	Animal	pST3Gal1	Gal(β1,3) GalNAcα	Rational point mutation and site-directed mutation library (mammalian surface display)	GT29	(Hombu et al. 2025)	Inactivated glycosyltransferase activity and increased binding to α2,3-sialyl core-2 O-glycan (H302A/A312I/F313S, sCore2)
DNA-binding protein	Bacterial	Sso7d	DNA	Site-directed mutation library (yeast surface display) and random mutagenesis	-	(Ward et al. 2023)	Increased binding to TF antigen and TF-antigen-containing carbohydrates (variant 1.3.D and 2.4.I)

#### Lectins found in nature

**Figure 2:**
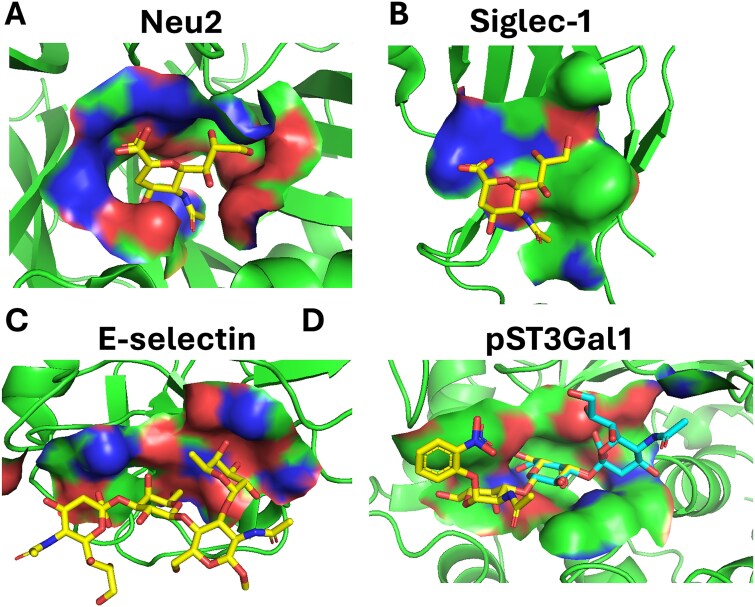
Binding interface of classic lectins vs. glycosyltransferases: a). Human sialidase Neu2 (PDB: 1VCU) bound to N-acetyl-2,3-dehydro-2-Deoxyneuraminic acid (DANA): 502Å^2^; b) mouse Siglec-1 (PDB: 1URL) bound to Neu5Acα-: 283Å^2^; c). Human E-selectin (PDB: 1G1T) bound to Neu5Acα2,3Galβ1,4[Fucα1,3]GlcNAcβ- (sLex): 401Å^2^; & d). pST3Gal1 (PDB: 2WNB) bound to Galβ1,3GalNAcα- overlayed with Neu5Acα2,3Galβ- (PDB: 5FRE): 542Å^2^_._ Protein backbone is shown as ribbon. Ligands are shown in stick form nested within the protein scaffold. Binding interfaces are shaded.

UniLectin3D has curated lectins based on the 3D structures of glycan-binding modules (Bonnardel et al. 2019). The surface area of these carbohydrate binding proteins is however relatively small, and thus these proteins often only recognize terminal monosaccharides or homopolymers, rather than more complex structures. For example, Siglec-1/−5/−7 have binding interfaces of 283–328 Å^2^; P- and E-selectin 325–401 Å^2^; and galectins 300–325 Å^2^, all of which are smaller than the binding interface of a glycosyltransferase like the α(2–3)sialyltransferases ST3Gal1 (542 Å^2^, Fig. 2).

**Figure 3:**
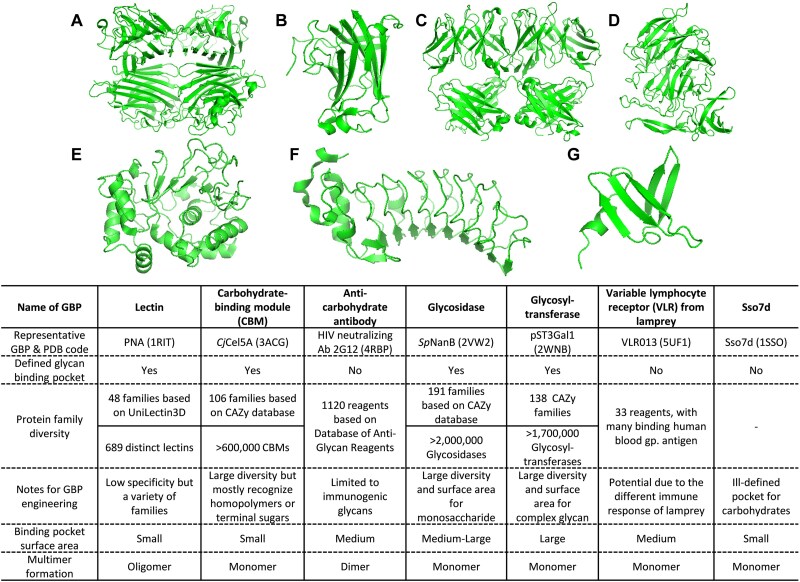
Scaffolds for GBP engineering: a) PNA, b) CjCel5A, c) mAb 2G12, d) SpNanB, e). pST3Gal1, f). VLRB, g). Sso7d.

Attempts have been made to tune the specificity of existing lectins to improve or expand their specificity using protein engineering (Fig. 3, Table 1, top section). In an early study, Drickamer showed that mutating Glu 185 to Gln 185 and Asn 187 to Asp 187 in the carbohydrate-recognition domain of a C-type mannose-binding protein switches its sugar-binding preference from mannose to galactose (Drickamer 1992). Adam et al. used rational engineering of PA-IIL lectin from the bacteria *Pseudomonas aeruginosa* to develop three single-site mutants by mutating the monosaccharide binding loop of the protein (Adam et al. 2007). They noted that S23A and G24N mutations increased binding to Me-α-L-Fuc compared to wild-type PA-IIL, while the S22A mutation increased binding to Me-α-L-Man. Another bacterial tandem repeat lectin, from *Pseudomonas taiwanensis* (PTL), has also been engineered for enhanced hemagglutination and antiviral activity (Matoba et al. 2021). Here, specific mutations were introduced in the weaker glycan-binding pocket (motif II, A54N, Q62R and Q74E) in order to mimic the stronger glycan-binding pocket in motif I. This resulted in higher anti-influenza activity for the mutant lectin compared to parent PTL. Swanson et al. introduced an H84T mutation in the loop region of BanLec that segregated two carbohydrate binding sites (Swanson et al. 2015). The mutation minimized lectin mitogenic properties (i.e immune cell stimulation) compared to wild-type protein while simultaneously retaining high antiviral potency. Thus, lectin binding engineering can benefit additional biological activities.

While the above cases use rational design for lectin engineering, others have used library-based approaches. In one example, the carbohydrate-recognition domain of *Maackia amurensis hemagglutinin* (MAH) was subjected to random mutagenesis while preserving the critical residues D127, H132, and D135 that bind sialic acid (Yim et al. 2001). This resulted in a panel of mutant lectins with diverse and enhanced hemagglutination activity towards erythrocytes from various animal species. Soga et al. successfully expanded the binding specificity of peanut agglutinin (PNA), which naturally recognizes the Thomsen-Friedenreich (TF) antigen, to also recognize sialyl-TF (NeuAcα2,6(Galβ1,3)GalNAc) (Soga et al. 2015). By employing rational site-directed mutagenesis libraries and mammalian cell surface display, the authors demonstrate an effective strategy for broadening the binding specificity of natural lectins. In another study, an N46A mutation in the fungal lectin, *Agrocybe cylindracea* galectin (ACG) improved binding specificity to glycans containing Forssman antigen (GalNAcα1,3Gal/GalNAc-), while eliminating binding to all other β-galactosides (Hu et al. 2013).

Among animal lectins, E-selectin was computationally reprogrammed to shift its specificity from sialyl Lewis-X antigen (sLe^X^) to 6′-sulfo-sLeX and 6′-sulfo-sialyl LacNAc, thereby mimicking the unique binding profile of Siglec-8 (Wang et al. 2022). By mutating key residues E92 and E107, which interact with the 6-OH group of galactose, the authors removed unfavorable positive charges from the binding pocket, allowing accommodation of 6′-sulfo-Gal and excluding the original sLe^X^ ligand. The stabilized, Cys-free Galectin-1 variant, CSGal-1, was engineered using non-canonical amino acid incorporation to alter its glycan binding specificity (Tobola et al. 2022). In this study, the incorporation of 7-fluorotryptophan at highly conserved W77 (CSGal-1[7FW]) modestly enhanced binding to ligands with terminal LacNAc, while reducing affinity for 3′-sulfo-LacNAc. In a final example, the worm lectin EW29Ch, which binds galactose-containing glycans, has been engineered both to recognize α2,6-Sia (Yabe et al. 2007; Hirabayashi and Arai 2019) and 6-sulfo-Gal (Hu et al. 2012). In this regard, EW29Ch has two glycan-binding domains: α-subdomain and γ-subdomain. Using random mutagenesis and selection using ribosome display, the authors introduced six mutations in the protein which imparted new sialic acid binding properties but reduced Gal dependence. Among the mutations, the G239S mutation in the α-subdomain resulted in new hydrogen bonding to the carboxylic acid of sialic acid, while D230G reduced the glycan-binding ability of the γ-subdomain. In an independent study, engineering E20K into EW29Ch resulted in a 6-sulfo-Gal-binding lectin, while additionally retaining the original Gal binding function (Hu et al. 2012). Overall, these studies present examples where the binding specificity of natural lectins has both been expanded to accommodate additional ligands and instances where new ligands are accommodated while losing the original specificity.

#### Carbohydrate-binding module (CBM)

CBMs commonly appear as part of glycosylating enzymes (glycoside hydrolases, polysaccharide lyases, and carbohydrate esterases), imparting non-catalytic glycan or substrate recognizing properties. This helps bring the catalytic domain of the enzyme in proximity to the substrate. For example, CBM40 from *Clostridium perfringens* contains a sialic acid-binding module that can function as a GBP (Ribeiro et al. 2016). Including tandem repeats to enhance avidity is a common strategy to enhance the otherwise weak binding affinity of such modules (Connaris et al. 2009; Ribeiro et al. 2016). All together the CAZy database reports 106 families of CBMs, that are categorized into three types: Type A CBMs recognize flat polysaccharide surfaces, Type B internal polysaccharide sites, and Type C glycan termini.

Efforts have been made to engineer CBMs, particularly for the purpose of efficient polysaccharide hydrolysis (Fig. 3, Table 1**, bottom section**). In one example, TlGa15B-GA2 (from *Talaromyces leycettanus*) was observed to express at lower levels compared to ReGa15A (from *Rasamsonia emersonii*) in yeast *K. phaffii* or *P. pastoris* (Tong et al. 2022), even though their glucoamylase activity was similar. Using domain swapping, segment replacement, and site-directed mutagenesis in the CBM, the authors identified key regions and specific combinations of amino acid mutations that significantly increased the expression and secretion of TlGa15B-GA2. Three engineered mutants (TlGA-M4, TlGA-M6, and TlGA-M7) showed 4–5 times higher enzyme activity and secretion levels compared to the wild-type enzyme. In another example, the linker and CBM of Cel7A (from *Trichoderma reesei*) was engineered to reduce non-productive binding to lignin thereby enhancing cellulase driven biomass hydrolysis (Strobel et al. 2016). The authors found that introducing negatively charged residues, such as the V27E mutation, improved selectivity for cellulose, while substitutions with hydrophobic or positively charged amino acids increased binding to lignin. Similarly, others demonstrate that supercharging the CBM by introducing multiple charged amino acid mutations without altering the catalytic domain can enhance the thermostability, binding affinity, and catalytic activity of cellulase from *Thermobifida fusca* (DeChellis et al. 2024). In this cellulase, positively charged CBM mutants showed increased binding to cellulose. In a final example, a combinatorial library was constructed by introducing mutations at 12 residues in CBM4–2, the xyloglucan-binding CBM from *Rhodothermus marinus* (Gunnarsson et al. 2004). CBM4–2 naturally exhibits broad binding specificity for xylans, β-glucans and cellulose. Using phage display, the authors selected CBM4–2 variants with new specificity for different carbohydrate polymers including birchwood xylan, Avicel and ivory nut mannan. These engineered CBMs retained high thermostability and could be produced at high yields in *E. coli*. Subsequent studies further characterized the CBM4–2 X-2 variant, confirming its specific binding to xylan (Cicortas Gunnarsson et al. 2007). Building on this work, the versatility of the CBM4–2 scaffold could be further expanded by applying random mutagenesis and selection to generate a variant with specific affinity for xyloglucan (von Schantz et al. 2009). These streamlined efforts to engineering CBM4–2 demonstrate the promise of modifying promiscuous CBMs for targeting increasingly complex polysaccharides. While most CBMs target carbohydrate homopolymer-degrading enzymes, unlike lectins that recognize complex glycans, these studies highlight the potential that engineering CBMs can advance biotechnology applications.

#### Anti-carbohydrate antibodies

Antibodies are essential components of adaptive immunity produced by B cells and plasma cells in response to foreign antigens. Antibodies feature variable Fab regions that recognize antigens, and constant Fc regions that mediate effector functions. The diversity of the Fab region, generated through somatic hypermutation, enables antibodies to recognize a vast array of foreign antigens. Despite the ability to generate diverse antigen-binding molecules, only non-self immunogens typically elicit antibody generation. Because many glycans are shared across species, human carbohydrates only elicit low immunogenicity in other mammals, posing challenges with the production of anti-carbohydrate antibodies. Despite this limitation, several anti-carbohydrate antibodies have been generated against tumor and blood group antigens, and microbial glycans (Haji-Ghassemi et al. 2015).

Engineering Fab and scFv (single-chain variable fragment) antibody fragments is one avenue to overcome the limitations of low-immunogenicity, self-recognition, and charged nature of carbohydrate antigens. For example, rational design was used to construct a human Fab phage display antibody library (FAB-CCHO) in which a short, six amino acid region of heavy chain CDR3 (complementary-determining region) was engineered to include basic (positively charged R, K) residues (Schoonbroodt et al. 2008). This design biased the binding pocket to interact with negatively charged carbohydrate antigens. This approach enabled the development of antibody fragments against sulfated sLe^X^ and heparan sulfate. Phage display is, however, not without challenges (Yuasa et al. 2010). Whereas these authors report the production of anti-carbohydrate antibodies against some antigen like LNFPIII (lacto-*N*-fucopentaose III), other scFv-Fc proteins were retained intracellularly and not well secreted. In another study, an yeast-display scFv library derived from ovarian cancer patients was used to select for tumor-associated bisecting N-glycans on the glycoprotein periostin in ovarian cancer (Lu et al. 2019). These authors used ovarian cancer cells overexpressing the target bisecting N-glycan, for positive selection and non-malignant cells for negative selection to enrich for the target glycan binding antibody. The resulting scFvC9 antibody was shown to target ovarian cancer cells and xenograft tumors in vivo, suggesting potential translational value. A further example involves affinity maturation of a previously developed anti-sialyl Lewis-a antibody (sLe^a^, 1116-NS-19-9) using yeast surface display to present the antibody, and error-prone PCR mutagenesis to mutate it (Amon et al. 2020). Here, nanoparticles bearing multivalent sLe^a^ were used as targets, and fluorescence-activated cell sorting (FACS) enabled selection of clones with enhanced binding. This approach yielded new anti-sLe^a^ clones with >70-fold increase in affinity.

The low immunogenicity of carbohydrate antigens limits the generation of anti-carbohydrate antibodies, and moreover antibodies against glycans frequently suffer from low binding specificity (Manimala et al. 2007). Nevertheless, it is important to note that glycan antigens have been injected into mammals to successfully generate antibody response, notably in the Phase I human trials by Danishefsky and colleagues where the immunogen was GloboH covalently linked to keyhole limpet hemocyanin (KLH) (Danishefsky et al. 2015). More recently, Gildersleeve presented studies where synthetic haptens, glycoproteins, and whole cells were injected into activation-induced cytidine deaminase (AID) knockout mice that lack somatic hypermutation machinery (DeLaitsch et al. 2022). The investigators report the generation of germline antibodies against an assortment of tumor-associated carbohydrate antigens, including Lewis Y, the Tn antigen, sialyl Lewis C, and Lewis X (CD15/SSEA-1).

#### Variable lymphocyte receptor (VLR) from lamprey

Analogous to immunoglobulin-based antibodies (Ig), jawless vertebrates such as lamprey and hagfish have developed Variable Lymphocyte Receptors or VLRs to function as antigen receptors. These VLRs are composed of multiple leucine-rich repeat (LRR) modules that form a horseshoe-shaped structure (Fig. 3, Table 1**, bottom section**). VLR diversity is generated by a gene conversion-like process, where LRR cassettes located in a single gene locus are randomly assembled to create different proteins (Pancer et al. 2004). Compared to ~200 glycosyltransferases found in the human and mouse genome, lamprey have only 128, suggesting that they may yield higher immunogenicity against GBPs of human relevance (McKitrick et al. 2020a). Consistent with this, the immunization of lamprey with whole mammalian cells, tissue homogenates and human milk resulted in VLRs that recognize carbohydrate antigens (McKitrick et al. 2020b). Using yeast surface display coupled with glycan microarray screening, VLRs that bind TF antigen (Luo et al. 2013), 3-sulfo-Gal (McKitrick et al. 2021), and Fucα1-2Galβ1–4Glc (Hong et al. 2013) were identified. Glycan-specific VLRs expressed as soluble IgG chimeric fusion proteins are now termed “smart anti-glycan reagents (SAGRs)” (McKitrick et al. 2020a). Such reagents can be used in place of antibodies in diagnostics and laboratory settings. These reagents can distinguish between differences in glycan structures, such as linkages, functional groups, and presentations (e.g. N-glycan vs. O-glycan). The difference in response of lamprey versus human to glycan immunogens, and their ability to generate VLRs against a wider swath of complex carbohydrate antigens remains under investigation.

#### Glycan-processing enzyme (glycosyltransferase and glycosidase)

Glycan-processing enzymes or glycoenzymes catalyze the addition or subtraction of monosaccharides or oligosaccharides from glycoconjugate scaffolds. These enzymes are well-curated, with the CAZy database currently listing 138 glycosyltransferase and 191 glycosidase families (Drula et al. 2022). Humans also have 400+ enzymes regulating glycosylation, including 247 transferases and 50 glycosidases (Groth et al. 2022). The diversity of this class of enzymes and distinct substrate specificity of each member makes them attractive scaffolds for engineering GBPs of biomedical relevance. In an early example, treatment of the influenza C hemagglutinin with the serine-esterase inhibitor diisopropyl fluorophosphate (DFP) selectively ablated viral esterase activity, thereby enabling the virus to specifically bind cells bearing 9-O-acetyl-N-acetylneuraminic acid (Neu5,9Ac₂) (Muchmore and Varki 1987). Thus, the DFP-treated virus serves as a specific probe for detecting 9-O-acetylated sialic acid.

Glycosidases commonly recognize terminal carbohydrate epitopes, contain well-formed binding pockets, and therefore exhibit high specificity (Fig. 3, Table 1**, bottom section**). In one example, bacterial sialic acid-processing glycosidases or sialidases have been engineered using iterative computation-guided directed evolution to develop pan-sialoglycan and more specific α2,3-sialoglycan binding proteins (Yang et al. 2002). More recently, additional groups have pursued similar goals (Liang et al. 2025). This study generated sialoglycan binding proteins by enzymatically inactivating sialidase activity in *Streptococcus pneumoniae* neuraminidase A (SpNanA) and *Ruminococcus gnavus* neuraminidase H (RgNanH). Here, mutating the catalytic acid residue D372 in *Sp*NanA resulted in a pan-sialic acid binding protein consistent with the broad enzyme activity of the starting protein, while modifying D282 in *Rg*NanH resulted in a α2,3-specific binder consistent with the specificity of the parent neuraminidase. Using these tools, the authors mapped the spatial distribution of sialoglycans in mouse organs and tissues, revealing distinct patterns of sialoglycan expression, especially in the intestine, heart, kidney, and lung.

**Figure 4:**
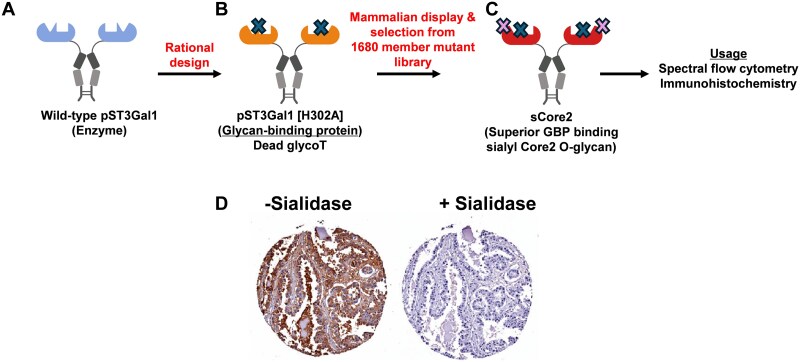
Engineering glycosyltransferases into glycan binding proteins. Conversion of pig ST3Gal1 (panel a) into glycan binding protein (GBP) using rational design (b) and mammalian surface-display mutation library screening (c). d) Staining of single ovarian cancer tumor tissue core (paraffin-embedded) with sCore2 GBP in the absence (left) and presence (right) of sialidase.

As glycosyltransferases have a larger binding interface and exhibit more precise substrate specificity compared to glycosidases, we determined if more specific GBPs can be developed by engineering the transferases (Hombu et al. 2025). The focus was on mammalian enzymes and sialylated epitopes due to their biomedical relevance (Hombu et al. 2021). To this end, porcine ST3Gal1 was employed as a scaffold and an H302A mutation was introduced in the donor (CMP-sialic acid) binding pocket (Fig. 4A-B). This resulted in a protein that bound the α2,3-sialyl core-2 O-glycan as determined using both a glycogene CRISPR knockout screen (Zhu et al. 2021; Kelkar et al. 2022) and glycan microarray analysis (Blixt et al. 2004). The resulting GBP, however, lost sialyltransferase activity. Additional library screening using a new Type-II mammalian surface display platform (Hombu and Neelamegham 2025), resulted in the development of an H302A variant called “sCore2” that exhibited exquisite binding for the sialyl core-2 epitope (Hombu et al. 2025) (Fig. 4C). Using this new GBP, we observed that sialylated core-2 O-glycans are highly expressed on selected myeloid lineages and also effector lymphoid populations, but not naïve cells. Using normal and cancer tissue microarrays, we also observed higher binding of sCore2 to selected proliferating tissue types including ovarian cancer samples (Fig. 4D).

Overall, the engineering of glycosidases and glycosyltransferases into GBPs provides a strategy to develop new binders based on the specificity of the original starting enzyme. This affords a predictive strategy to develop entire new classes of GBPs using rational “design” as a first step, followed by high-throughput screening for optimization.

### Non-glycan binding scaffolds

While many of the above scaffolds have a natural ability to bind glycans, attempts have also been made to engineer GBPs from scaffolds that do not naturally recognize carbohydrate antigens (Fig. 3, bottom of Table 1). For example, attempts were made to modify the archaeal DNA-binding protein Sso7d to recognize TF antigen and related structures (Ward et al. 2023). After two rounds of affinity maturation, the best variant (2.4.I) indeed showed low micromolar affinity for TF, comparable to commercially available lectins. However, it also showed binding to other structurally related or even distinct glycans (e.g. polysialic acid, chitobiose). Others have determined if aptamers, short nucleotide chains, can be evolved to bind carbohydrates. A popular strategy uses the systematic evolution of ligands by exponential enrichment (SELEX) method, where randomly synthesized oligonucleotides are subjected to selection against the specific ligands. These are amplified and sequenced, and then additional rounds of evolution proceed for aptamer binding maturation. During the synthesis of oligonucleotide libraries, it is also feasible to incorporate non-canonical nucleotides to increase molecular diversity. Several glycoprotein-specific aptamers have been identified using non-canonical nucleotides such as boronic acid (Li et al. 2008) and indole (Yoshikawa et al. 2021). With boronic acid, a fibrinogen-specific aptamer was identified. Indole-modified aptamers evolved to recognize glycosylated RNase B and fetuin. The RNase B-specific aptamer was found to bind mannosylated N-glycan. In additional developments, aptamers targeting human prostate-specific antigen (hPSA) (Diaz-Fernandez et al. 2020), high-mannose (Li et al. 2023), and Neu5Ac (Yue et al. 2021) have been described. Although the negative selection using un-glycosylated target proteins is performed in these reports, it is not known whether the glycosylated proteins used in the positive selection have well-defined glycans, as this can impact aptamer binding specificity. Nevertheless, the large library size of aptamers is attractive for creating diversity. More studies are needed to determine if non-glycan scaffolds can yield specific GBPs.

### Directed evolution for glycan binding protein engineering

As shown in the previous section, methodologies used for lectin engineering are often limited to traditional site-directed mutagenesis. State-of-the-art directed evolution strategies can potentially drive greater innovation. This section discusses the potential for such schemes with focus on: (i) Cell surface display to enable high-throughput screening of diverse mutant libraries, and (ii) Newer mutagenesis strategies for in situ GBP engineering.

#### Cell surface display

Cell surface display is a key driver of the protein evolution field as this establishes the link between target gene expression/sequence in individual cells and corresponding function assayed on the surface. This could be used for studying both carbohydrate dependent binding and glycoenzyme activity. The power of this approach was first demonstrated using phage display, a technology now commonly used to aid antibody and protein design (Smith 1985; Mccafferty et al. 1990). Ricin was the first lectin to be displayed on the phage (Swimmer et al. 1992). Phage display is advantageous due to the large size of mutant libraries that can be expressed (10^10^–10^12^) and fast replication speed. To study the vast number of carbohydrate-binding domains expressed in the human gut microbiota, in one study, phage display was employed and novel carbohydrate-binding domains were identified (Akhtar et al. 2023). This demonstrates the potential to discover new GBPs with novel structures. Although phage display is suitable for screening bacterial lectins, it may be challenging to display mammalian lectins on phage. To overcome this limitation, SpyCatcher-SpyTag conjugation chemistry has been applied to capture mammalian lectins such as Siglec-7 on phage (Lima et al. 2024), though mammalian protein mutagenesis and screening is not possible using this approach. Thus, phage can be used as the carrier for increasing the multivalency of any lectins. Phage can display a portion of human antibodies such as scFv and Fab, and it has also been applied to discovery therapeutic agents (Zhang 2023). Supporting the use of this platform in the glycosciences, indeed, scFv-displaying phage display libraries have been used to discover proteins that recognize tumor-associated carbohydrate antigens such as sialyl Lewis-X and Lewis-X (Mao et al. 1999).

Whereas phage does not allow the display of large proteins due to its small size (10 ~ 100 nm), bacteria (~ 1μm size) can overcome this limitation. The molecular library size of bacteria also rivals phage and the replication rate is high. Indeed, human lectins such as Galectin-3 and mannose-binding lectin (MBL) have recently been displayed on the bacterial cell surface using *N*-terminal membrane-anchoring region of intimin (Vazquez-Arias et al. 2024). Despite this success, it is quite challenging to express most human lectins in bacterial hosts due to different protein post-translational modification machineries and the lack of endoplasmic reticulum (ER) and Golgi. Thus, solutions in phage and bacteria may not be generalizable across mammalian lectins. Other than lectins, bacterial sialyltransferases have also been displayed on the bacterial cell surface by tethering the enzyme at the C-terminus of outer membrane porin C (OmpC), Thus, examples in literature suggest that bacterial display can be used to engineer glycan-processing enzymes and lectins at least for some classes of proteins (Song et al. 2019).

Although the bacterial system is beneficial for creating large libraries, human proteins expressed in prokaryotes often appear in inclusion bodies and this may bias the expression of mutant libraries. To address this shortcoming, yeast display has been used for screening eukaryotic proteins (Boder and Wittrup 1997). In this regard, yeast can host large mutant libraries (Zhang et al. 2020). It is also a single-cell eukaryote with an ER and Golgi, which are pivotal for folding of functional mammalian proteins. Due to this, yeast display may serve as an intermediate host for screening studies utilizing both bacterial and mammalian lectins. The most frequently used platform for yeast surface display is the α-agglutinin, Aga1p-Aga2p system, where Aga1p is displayed on the cell surface and Aga2p is fused to *N*- or *C*-terminus of the protein of interest along with disulfide bond linkage to Aga1p (Uchanski et al. 2019). This platform as well as the other glycosylphosphatidylinositol (GPI)-anchored α-agglutinin, SAG1, can be used to display at least some human lectins including galectins and sialoadhesins (Ryckaert et al. 2008). These surface-displayed lectins are functional in that they bind glycosylated polyacrylamide (PAA)-polymers. The platform has also been used to identify proteins with carbohydrate binding affinity from complex metazoan cDNA libraries using FACS for enrichment. Although human lectins can be displayed on yeast, attention needs to be paid to protein hyper-mannosylation in these cells, which could inhibit protein-carbohydrate interactions and enzyme activity (Luley-Goedl et al. 2016). Thus, OCH1 mannosyltransferase-deficient yeast strains and GlycoSwitch variants that produce human-like carbohydrates may be preferred if hyper-mannosylation is a concern (Jacobs et al. 2009; Li et al. 2022).

**Figure 5:**
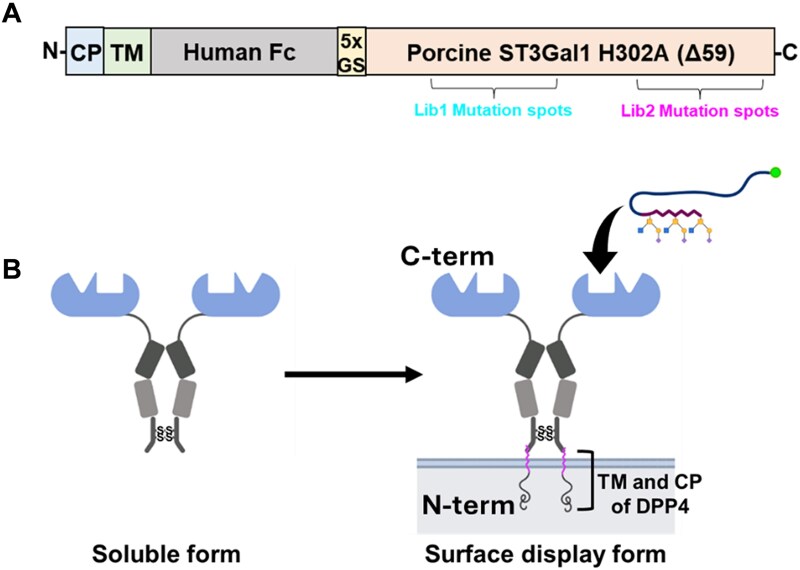
Surface display of glycan binding protein derived from pig ST3Gal1: **a**) plasmid construct for expression of type-II membrane proteins contains cytoplasmic (CP) and transmembrane region (TM) of DPP4 followed by human IgG1 fc, flexible Gly-ser linker and truncated enzyme lacking the N-terminal cytoplasmic and transmembrane regions (pST3Gal1Δ59). **b**) Expression of GBP on cell surface and screening using glycans presented on polyacrylamide-based fluorescent polymer.

Mammalian cells as a host for surface display are an attractive choice, particularly for studies of human biology. Such systems are commonly used to present chimeric antigen receptors (CARs) in immune cells in the context of immunotherapy. Here, the extracellular domain (often scFv) interacts with the antigen receptor on the target cells, with the transmembrane domain anchoring the CAR on cells, and intracellular domain triggering the immune cells. In most cases, such constructs are Type-1 membrane proteins with N-terminus outside cells (Park et al. 2024). In an interesting study, peanut agglutinin has been presented in CAR-like constructs with the N-terminal lectin followed by a natural killer cell p46-related protein stalk, CD8α transmembrane domain, and intracellular signaling domain containing CD3ζ that triggers a GFP reporter. (Soga et al. 2015). This system enabled the screening of a mutant library of PNA, determining key loop C residues that are critical for sugar-binding specificity and affinity. As opposed to traditional CAR proteins, glycosyltransferases presented in the Golgi are Type-II membrane proteins with the N-terminus facing the cytoplasm. Type-II membrane proteins are less common on cell surfaces. In a recent study, we developed a robust Type-II membrane presentation strategy (Fig. 5). Here, we presented both sialyltransferases (Hombu and Neelamegham 2025) and sialyltransferase-derived GBPs (Hombu et al. 2025) on mammalian cell surfaces by anchoring the enzyme fused to human IgG1 Fc and Type-II transmembrane domains from either DPP4 or CD94. This platform helped screen ~1680 mutations in lectins and enzymes to discover both new GBPs that bind sialyl core-2 glycans and new “super enzymes” with higher activity compared to native porcine ST3Gal1. While advantageous in terms of protein expression, library size in mammalian surface display is usually smaller (10^7^ ~ 10^9^) compared to bacteria and yeast.

Virus display is a potential option that can overcome the limitations of relatively small library size in mammalian cells and improper protein folding in prokaryotes. Although there are no examples of using viruses for the purpose of high throughput GBP engineering, viruses commonly express lectins that infect mammalian cells. For example, hemagglutinin (HA) on influenza binds sialoglycans on host, and modification of the HA sequence modifies cell tropism (Rogers and Paulson 1983; Liang et al. 2023). Virus pseudotyping has been done for adenovirus and lentivirus to direct the binding of virions to specific target cells (Frank and Buchholz 2019; Yang et al. 2020; Yang et al. 2022), and recent studies show that this can also be applied to virus-like particles where the surface glycoprotein can be readily swapped (Yang et al. 2025). In a final example, it is demonstrated that camelid antibodies can readily replace the knob domain of fiber capsid proteins of adenovirus to retarget the virus (Lee et al. 2021), and we envision that similar approaches may be feasible using lectin-library display on adenoviruses.

In summary, surface display is an essential tool for GBP engineering. The selection of platforms depends on many considerations including source of the starting lectin, post-translational modifications needed for its folding, library size, and host glycosylation status that may affect GBP function.

#### In situ *mutagenesis*

**Figure 6:**
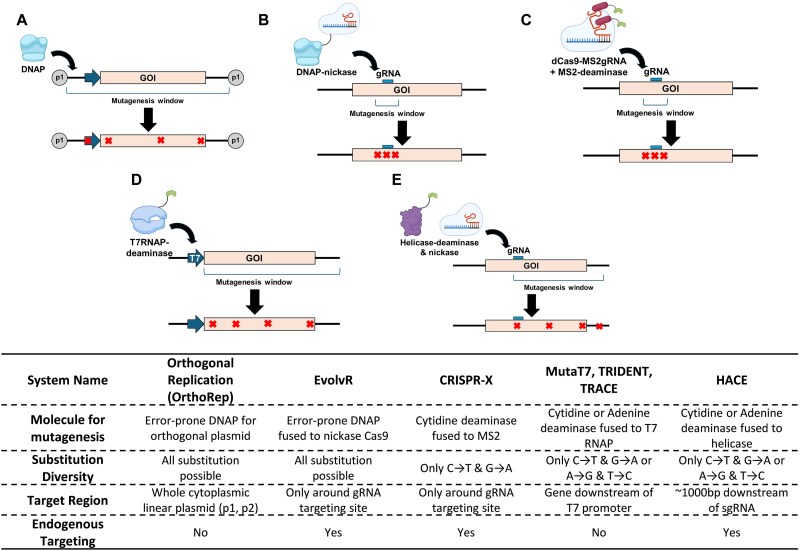
In vivo mutagenesis methods for glycan binding protein engineering. a) OrthoRep, b) EvolvR, c) CRISPR-X, d) T7RNAP-based, e) HACE.

Site-directed mutagenesis and in vitro random mutagenesis are widely used for lectin engineering. While these methods readily introduce mutations in target regions, in vivo mutagenesis is an attractive alternative. Such methods preclude the need for cloning mutagenized plasmids, transformation, and isolation of genes of interest after selection in each round (Molina et al. 2022). This section describes these state-of-the-art in vivo mutagenesis methods, and potential applications that can lead to the directed evolution of novel GBPs (Fig. 6).

In vivo error-prone DNA polymerase in host cells is an option for directed evolution. This platform was first developed in yeast by introducing orthogonal error-prone DNA polymerase outside the nucleus along with target linear plasmid (Ravikumar et al. 2014). In this “OrthoRep” system the error-prone DNA polymerase is localized in the cytoplasm, introducing all possible mutations including transversions on the linear target. Exceptional mutation rates (~ 10^−4^ substitutions per base) are achieved using this engineered DNA polymerase, one million times higher compared to natural genomic mutation rates (Ravikumar et al. 2018; Rix et al. 2024). OrthoRep can be used for engineering antibody fragments in combination with yeast surface display, implying that a similar approach may also be feasible for GBP engineering (Wellner et al. 2021; Paulk et al. 2024). This orthogonal replication platform was recently expanded to bacteria (Tian et al. 2023; Tian et al. 2024) and mammalian cells (Ma and Lin 2025). A similar approach is used in “EvolvR” where a programmable DNA-targeting protein (a Cas9 nickase) is linked to a low-fidelity DNA polymerase. Guided by a user-defined gRNA, EvolvR introduces mutations specifically at a chosen genomic locus in living cells in both bacterial and mammalian cells (Halperin et al. 2018; Hurtado et al. 2025).

Nucleoside deaminase-based mutagenesis is another method for in vivo random mutagenesis. Originally, cytidine and adenine deaminase were used individually to introduce a specific substitution to the target gene loci by fusing these enzymes to nickase Cas9 (Komor et al. 2016; Gaudelli et al. 2017). This platform has since been improved by combining the two deaminases to enable both cytosine and adenine editing (Grunewald et al. 2020; Sakata et al. 2020; Zhang et al. 2020), engineering Cas9 to expand the compatibility to target a greater number of genomic loci (Miller et al. 2020; Walton et al. 2020), and allowing transversions not possible by conventional base editors (Tong et al. 2023). Although this platform is attractive for correcting disease-relevant point mutations, GBP engineering requires the editors to create multiple mutations in a random manner across the gene to facilitate molecular evolution. To facilitate this, the CRISPR-X system was developed wherein a hyperactive variant of the activation-induced cytidine deaminase (AID*Δ) was fused to the MS2 bacteriophage coat protein that binds short MS2 RNA hairpin stem-loops with high specificity (Hess et al. 2016). Due to the design, multiple deaminases were recruited to dCas9 complexed with MS2 hairpin-sgRNA, and this enabled random mutations around the sgRNA targeting region. This approach has been used for antibody generation and thus can likely also be repurposed for GBP engineering (Devilder et al. 2019). Despite high-fidelity targeting, editing due to both CRISPR-X and EvolvR are limited to the narrow region proximal to the sgRNA recognition site. This limitation can be overcome by fusing nucleotide deaminase to T7 RNA polymerase, which targets the T7 promoter and edits the whole gene downstream of the promoter. This T7 RNA polymerase-based evolution tool has been used in bacteria (Moore et al. 2018; Cravens et al. 2021), yeast (Huang et al. 2023), and mammalian cells (Chen et al. 2020). Although long range mutagenesis is achieved, the cloning of a T7 promoter upstream of the target gene is necessary. To overcome this limitation so that endogenous genes can be targeted, the HACE (helicase-assisted continuous editing) system has been developed (Chen et al. 2024). Here, a Cas9 nickase is used to expose a genome region of interest in a guide-RNA directed manner. A DNA helicase-deaminase fusion protein loads onto this target loci, initiating long range mutagenesis downstream of the target site. Overall, there are active, exciting developments for in vivo directed evolution with new targeting strategies and base editors offering novel opportunities for GBP engineering.

## Conclusions

This review examines scaffolds that can serve as GBPs, and methods to optimize their design using protein engineering. It documents progress made in recent years, including the increasing relevance of protein engineering to the glycosciences field.

Among the scaffolds, although lectins are attractive starting material due to their natural glycan binding properties and wide availability, they commonly exhibit broad binding specificity due to their small binding interface, and low affinity which contributes to their promiscuity. Lectin engineering promises to address some of these limitations but challenges remain as lectins engineered to bind a new carbohydrate epitope must ideally also simultaneously lose binding to the original target. The CBM engineering examples highlight the suitability of this approach for biotechnology applications particularly with respect to polysaccharide-processing, but the ability of these scaffolds to detect complex carbohydrate epitopes remains to be established. The repertoire of anti-carbohydrate antibodies is currently limited due to the low immunogenicity of many carbohydrate epitopes. This is somewhat resolved by using non-mammalian hosts like lamprey due to differences in glycosylation pathways across species, but the ability of this resource to generate GBPs against arbitrary complex carbohydrate structures is not yet established. The ability of non-glycan-binding proteins to transform into specific GBPs also remains under study. Engineering of glycosidases and glycosyltransferases represents an emerging, promising route that could potentially result in “GBPs by design” based on the specificity of the starting entities. While examples of such engineering have only recently emerged in literature, this could represent a strategy that can eventually quantify the glycome on single cells.

In addition to scaffold design, modern technologies are needed for high-throughput GBP engineering. While error-prone PCR and more focused libraries have been used previously, newer techniques are coming online that are likely to transform the field. These technologies emphasize surface display for high-throughput screening of lectins and related scaffolds, and directed evolution for in situ mutagenesis. With respect to the former, traditional surface display technologies for lectin engineering have evolved from classical bacteriophage, bacteria and yeast platforms to also include mammals in recent years. Important among these are the method to present Type-II transmembrane proteins on cell surfaces for glycosyltransferase engineering. This is particularly relevant to emerging interest in transforming glycoenzymes (which are often Type-II membrane proteins) into GBPs. Considering the in vivo mutagenesis schemes, low fidelity DNA polymerase-based mutagenesis is an important tool, as highlighted in examples of OrthoRep that have been successfully used for antibody engineering, raising the possibility that similar approaches could be applied to lectins. Deaminase-based mutagenesis is also an emerging area for base-editing, but transition mutations are favored by deaminase and this only results in limited molecular diversity compared to DNA polymerase-based mutagenesis. As new developments appear in this area, applications in GBP engineering will likely emerge. These efforts would be accelerated if selection strategies conferred a glycan-dependent growth advantage, wherein only cells expressing selected lectin-carbohydrate interactions survive.

Finally, while we noted many GBPs in our literature survey, a practical challenge relates to scalability and availability to the scientific community at low cost. The time seems ripe to require scientists to deposit GBP sequence data at a central repository and provide scaffolds so that the products of science are more accessible. This could serve as an important driver of glycobiological discovery.

## Data Availability

All data are incorporated in the article text.
